# To the Editors of the Medical and Physical Journal

**Published:** 1811-11

**Authors:** 

**Affiliations:** Golden Square


### To the Editors of the Medical and Physical Journal.

Gentlemen,

It appears, by recent accounts from France, that the sale of Quack-medicines has been prohibited in that country; the French government having, with a laudable policy, bought up the secrets of those remedies which were found to possess efficacy, and entirely suppressed those that proved to be noxious or inert. Now, I cannot help suspecting that the noted *Eau Medicinale d'Husson* ranked in the proscribed class; and in this conjecture I have been in some measure confirmed by the circumstance of a *transfer* of the sole right to vend the medicine having been made by the proprietor, M. Chardron, to his agent in this country, much about the time that the above-mentioned suppression of secret nostrums is said to have taken place. Perhaps some of your correspondents may be able to furnish further information on this subject.

I am, Gentlemen,

A QUERIST.

*Golden Square, Oct.* 17.

---

